# HAPPY IN MY HOME AND COMMUNITY: PREFERENCES OF OLDER RURAL RESIDENTS

**DOI:** 10.1093/geroni/igz038.1298

**Published:** 2019-11-08

**Authors:** Joanne V Binette

**Affiliations:** AARP, Washington, District of Columbia, United States

## Abstract

AARP will introduce research on rural residents from the 2018 Home and Community Preferences Survey of Adults Age 18 and Older that shows the importance of making communities places where rural residents can successfully age throughout all stages of life. This data will provide useful insights regarding what adults who live in rural areas want and need in their communities to positively contribute to their overall health and well-being and keep them actively engaged and involved in their community. The survey data can serve as a key tool for communities to understand what rural residents view as important for successfully aging in place and strategies that communities might employ to create community supports and services conducive for older rural residents.

